# Effects of music and conversation on pain and anxiety levels during transrectal ultrasound-guided prostate biopsy: a randomized-controlled prospective study

**DOI:** 10.1590/1516-3180.2025.3273.28072025

**Published:** 2025-12-15

**Authors:** Volkan Selmi, Mehmet Sakir Taspinar, Mehmet Caniklioglu, Sercan Sari, Mahmut Akyuz, Levent Isikay

**Affiliations:** I Urological surgeon; Associate Professor, Faculty of Medicine, Urology Department, Yozgat Bozok University, Yozgat, Türkiye.; II Urological surgeon; Assistant Professor, Faculty of Medicine, Urology Department, Yozgat Bozok University, Yozgat, Türkiye.; III Urological surgeon; Associate Professor, Faculty of Medicine, Urology Department, Yozgat Bozok University, Yozgat, Türkiye.; IV Urological surgeon; Associate Professor, Faculty of Medicine, Urology Department, Yozgat Bozok University, Yozgat, Türkiye.; V Psychiatrist, Department of Psychiatry, Afsin State Hospital, Kahramanmaras, Türkiye.; VI Urological surgeon; Professor, Faculty of Medicine, Urology Department, Yozgat Bozok University, Yozgat, Türkiye.

**Keywords:** Prostate, Biopsy, Anxiety, Pain, Ultrasound, high-intensity focused, transrectal, Prostate biopsy, Anxiety, Pain, Coversation, Music

## Abstract

**BACKGROUND:**

Prostate biopsy is the recommended diagnostic test for prostate cancer in patients with abnormal findings on digital rectal examinations (DRE) or elevated PSA (prostate-specific antigen) levels. Biopsies can cause severe anxiety and pain.

**OBJECTIVE:**

To evaluate the effects of music and conversation on pain and anxiety in patients undergoing transrectal ultrasound-guided prostate biopsy.

**DESIGN AND SETTING:**

Prospective trial at a Tertiary University Hospital in Türkiye.

**METHODS:**

A prospective randomized controlled study was conducted. Ninety patients who had abnormal findings on DRE and/or a PSA value greater than 2.5 ng/ml and were scheduled to undergo TRUS-PBx were randomly assigned to one of three groups (n = 30) via the sealed envelope randomization method: control, music, and conversation groups. VAS pain, VAS anxiety, and STAI scores were recorded before and after the procedure. Statistical analysis was performed using One-Way ANOVA and Kruskal–Wallis tests. Pairwise comparison tests were conducted for the parameters that yielded significant differences among the control, conversation, and music groups. For all statistical analyses, a P value < 0.05 was considered statistically significant.

**RESULTS:**

Conversation reduced pain and anxiety significantly (P < 0.001 and P = 0.01 respectively). Post-Bx VAS pain and STAI-State scores were lower in the conversation group. Pairwise comparisons revealed significant differences between the music and conversation groups (VAS pain, P < 0.001; STAI-State, P = 0.006). However, pain and STAI-State scores were similar in both the groups (VAS pain P = 0.645; STAI-State P = 0.597).

**CONCLUSIONS:**

The study demonstrated that listening to music had no significant effect on pain and anxiety in patients undergoing TRUS-PBx. Conversely, the findings showed that engaging patients in conversation significantly reduced pain and anxiety during the procedure.

**CLINICAL TRIAL OR SYSTEMATIC REVIEW REGISTRATION:**

The trial is registered at clinicaltrials.gov (registration number: NCT07006779) and is accessible at https://clinicaltrials.gov/ct2/show/NCT07006779.

## INTRODUCTION

 Prostate cancer is one of the most common cancers affecting men. Prostate biopsy is the recommended diagnostic test for prostate cancer in patients with abnormal findings during digital rectal examination (DRE) or elevated prostate-specific antigen (PSA) levels.^1^ Although transperineal prostate biopsy is increasing, standard transrectal ultrasound-guided prostate biopsy (TRUS-PBx) shows similar prostate cancer detection rates.^2^ When the procedure is explained, patients with no prior experience are particularly susceptible to anxiety.^3^ Moreover, increased anxiety during the procedure is associated with higher pain perception and irritation. Therefore, biopsy procedures can cause intense anxiety and severe pain for patients.^4^

 Since the biopsy procedure is performed under local anesthesia, anxiolytic or sedative drugs are rarely administered.^5^ Patients who experience severe pain during the intervention often avoid undergoing re-biopsy when necessary.^6^ For these reasons, numerous studies and interventions have been conducted to reduce pain and anxiety in patients undergoing TRUS-PBx. A wide range of methods has been evaluated, from general anesthesia to combinations of local anesthesia with other techniques. Many nonpharmacological interventions, including distraction and anxiety-pain reduction techniques, have been investigated because of their practicality, accessibility, convenience, and minimal side effects.^7-10^

 Music reduces pain, anxiety, and stress. Its effectiveness is attributed to its ability to divert patients’ attention away from negative stimuli and create a calm environment.^11,12^ The positive effects of music on patients undergoing TRUS-PBx have also been evaluated in several studies.^13-19^

 Conversation is another effective distraction method that is free, easily accessible, and has no side effects. As anxiety levels increase, the pain experienced by patients also tends to increase. Consequently, reducing patients’ anxiety through conversation can lower the pain level.^20^ Reportedly, conversing with patients can reduce the anxiety and pain they experience.^20-26^ However, there is limited research on the impact of conversation in reducing pain, anxiety, and stress, specifically in patients undergoing prostate biopsy. 

 This is the first prospective randomized controlled study to investigate the effects of listening to music or engaging in conversation on procedure-related anxiety and pain in patients undergoing TRUS-PBx. 

## OBJECTIVE

 To evaluate the effects of music and conversation on pain and anxiety levels in patients undergoing TRUS-PBx. 

## METHODS

### Study design and population

 This prospective randomized controlled study was approved by the Local Clinical Research Ethics Committee (2017KAEK-189-2021.05.05-08). The clinical trials. gov registration number for the trial is NCT07006779. Patients included in the study were those who presented to the Yozgat Bozok University Hospital Urology Clinic between June 2021 and October 2021, had abnormal findings on DRE and/or had a PSA value greater than 2.5 ng/ml and were scheduled to undergo TRUS-PBx. Sample sizes were calculated using the program GPower v.3.1.9.4 (Franz Faul, University of Kiel) with an alpha value of 0.05, 95% confidence interval (95% CI) power, and 0.19 effect size. Ninety patients were randomly assigned to one of three groups (n = 30) using the sealedenvelope randomization method: control, music, and conversation groups. An experienced urologist generated a randomized allocation sequence, enrolled the participants, and assigned them to the interventions. An experienced urologist performed all procedures. 

### Inclusion and exclusion criteria

 Patients with psychiatric disorders, those who declined to participate, and those who were unable or unwilling to complete the questionnaires were excluded. Additionally, patients with hearing or perceptual impairments, continuous analgesic use, history of prior prostate biopsy, or prilocaine allergy were excluded. Patients for whom the biopsy procedure could not be completed for any reason were excluded. Informed consent was obtained from all the participants. 

### Data collection and definitions

 Patients’ sociodemographic data such as age, height, weight, and body mass index were documented. Medication and medical histories were recorded. The International Prostate Symptom Score (IPSS), PSA levels, and prostate volumes of all patients were recorded before the procedure. The blood pressure and heart rate were measured before and after the procedure. 

 The patients completed the State-Trait Anxiety Inventory (STAI) test. Low scores indicate low anxiety levels, whereas high scores reflect higher anxiety levels. Also, anxiety and pain levels were assessed using a visual analog scale (VAS), where a score of "0" indicated "no pain or anxiety," and a score of "10" represented "the most severe pain or anxiety." 

 Prophylactic ciprofloxacin was administered to patients 1 day before the procedure. The procedure room was designed to minimize external disturbances (e.g., sound or noise). Prior to biopsy, the patients received detailed information according to their assigned groups. In the music group, patient-selected music was played throughout the procedure. In the conversation group, discussions based on patients’ interests were initiated and continued during the procedure. In the control group, no additional interactions beyond the informational briefings were provided. 

 The biopsy procedure was performed with patients in the lateral decubitus position with their lower extremities flexed. Rectal povidone-iodine was used for local cleaning. Lidocaine gel was applied rectally in all patients. After placing the rectal ultrasound probe, a periprostatic nerve block was performed by injecting 5 ml of 2% prilocaine solution. Twelve core biopsies were collected: six from each lobe. 

 All patients in the allocated groups underwent TRUS-PBx as described previously. After the procedure, the patients were asked whether they would be willing to undergo the procedure again if necessary. Responses were categorized as "positive," "negative," or "abstained." 

### Primary and secondary outcomes

 The primary outcome of the study was to determine whether listening to music or engaging patients in conversation during the procedure reduced their anxiety and pain levels. The secondary outcome was to determine which was better for decreasing anxiety and/or pain levels: listening to music or engaging in conversation. 

### Statistical analysis

 Statistical analyses were performed using the IBM SPSS Statistics version 26.0 (Armonk, New York). Nominal data were analyzed using the Chi-square test. Parametric numerical data were evaluated using the One-Way ANOVA variance, whereas non-parametric data were analyzed using the Kruskal–Wallis test. Pairwise comparisons were performed to identify significant differences among the control, conversation, and music groups. Pre- and post-procedure comparisons were also performed within each group. For all analyses, a P value < 0.05 was considered statistically significant. 

## RESULTS

 Between June 2021 and October 2021, 101 patients at Yozgat Bozok University Training and Research Hospital Urology Clinic, were assessed for eligibility; however, 10 patients were not eligible and one declined to participate. Thus, 90 patients were randomly assigned to three groups (n = 30 each). Mean age and BMI were not significantly different among the groups (P = 0.339 and P = 0.383, respectively). Additionally, the mean IPSS, PSA value, and prostate volume were statistically similar among the three groups (P = 0.591, P = 0.582, and P = 0.262, respectively). The patient demographic data are shown in **Table 1**.

**Table 1:** The demographic data of patients

**Variables**	**Total (M ± SD) (n = 90)**	**Control (M ± SD) (n = 30)**	**Music (M ± SD) (n = 30)**	**Conversation (M ± SD) (n = 30)**	**P value**
Age ± SD, years	65.32 ± 7.47	64.5 ± 7.37	66.96 ± 7.9	64.5 ± 7.08	0.339
BMI ± SD, kg/m^2^	28.66 ± 3.18	29.29 ± 3.29	28.08 ± 3.03	28.61 ± 3.21	0.383
IPSS ± SD	17.58 ± 8.25	16.5 ± 8.84	17.53 ± 8.09	18.7 ± 7.91	0.591
PSA ± SD, ng/mL	13.4 ± 19.62	9.71 ± 6.7	18.43 ± 28.11	12.07 ± 17.45	0.582
Prostate volume ± SD, cc	68.69 ± 36.4	64.05 ± 42.06	70.2 ± 26.33	71.83 ± 39.60	0.262

M, mean; SD, standard deviation; BMI, body mass index; IPSS, International Prostate Symptom Score; PSA, prostate-specific antigen.

 Hemodynamic parameters, such as systolic and diastolic blood pressure and heart rate measured before biopsy, showed no significant differences among the three groups (P = 0.619, P = 0.684, and P = 0.723, respectively). Additionally, the pre-procedure VAS anxiety (P = 0.299), STAI (P = 0.560), STAI-State (P = 0.850), and STAITrait (P = 0.515) scores were similar across the groups (**Table 2**). 

**Table 2:** Comparison of pre-biopsy variables between the groups

**Variables**	**Total (M ± SD) (n = 90)**	**Control (M ± SD) (n = 30)**	**Music (M ± SD) (n = 30)**	**Conversation (M ± SD) (n = 30)**	**P value**
Pre-Bx Systolic Blood Pressure ± SD, mmHg	117.33 ± 15.96	117.5 ± 19.03	116.17 ± 14.72	118.33 ± 14.16	0.618
Pre-Bx Diastolic Blood Pressure ± SD, mmHg	70.75 ± 11.36	73.1 ± 14.31	69.5 ± 10.2	69.67 ± 8.9	0.684
Pre-Bx heart rate ± SD, bpm	80.64 ± 6.83	80.53 ± 8.06	80.1 ± 5.25	81.3 ± 7.06	0.723
Pre-Bx STAI ± SD	73.8 ± 16.77	71.63 ± 16.2	74.27 ± 18.63	75.5 ± 15.67	0.56
Pre-Bx STAI-State ± SD	34.1 ± 9.41	33.63 ± 8.93	34.2 ± 11.06	34.47 ± 8.34	0.85
Pre-Bx STAI-Trait ± SD	39.49 ± 9.88	37.9 ± 8.77	39.73 ± 10.62	40.83 ± 10.26	0.514
Pre-Bx Anxiety (VAS) ± SD	3.2 ± 1.7	3.03 ± 1.73	3 ± 1.23	3.57 ± 2.03	0.299

M, mean; SD, standard deviation; bx, biopsy; STAI, State-Trait Anxiety Inventory; VAS, visual analog scale; bpm, beats per minute.

 No statistically significant differences were found in the post-biopsy blood pressure or heart rate among the three groups (P = 0.284 and P = 0.355, respectively). The scores of VAS pain, VAS anxiety, and the STAI-State recorded after the biopsy procedure were significantly lower in the conversation group (P < 0.001, P < 001 and P = 0.010, respectively). 

 Statistically significant decreases in STAI-State scores, which were recorded before and after the biopsy, were observed only in the conversation group, whereas the scores increased in the other groups (P < 0.001) (**Table 3**). 

**Table 3:** Comparison of post-bx variables between the groups

**Variables**	**Total (M ± SD) (n = 90)**	**Control (M ± SD) (n = 30)**	**Music (M ± SD) (n = 30)**	**Conversation (M ± SD) (n = 30)**	**P value**
Post-Bx Systolic Blood Pressure ± SD, mmHg	115.11 ± 15.51	112.83 ± 17.8	115.17 ± 14.05	117.3 ± 14.61	0.284
Post-Bx Diastolic Blood Pressure ± SD, mmHg	68.77 ± 10.2	69.83 ± 10.87	68.5 ± 9.66	68 ± 10.31	0.767
Post-Bx heart rate ± SD, bpm	80.36 ± 4.83	81.13 ± 4.09	79.37 ± 4.68	80.57 ± 5.59	0.355
Post-Bx STAI ± SD	74.54 ± 15.9	77.77 ± 16.28	76.5 ± 16.99	69.37 ± 13.4	0.066
Post-Bx STAI-State ± SD	35.33 ± 9.54	37.87 ± 8.93	36.87 ± 11.33	31.27 ± 6.72	**0.010**
Post-Bx STAI-Trait ± SD	38.95 ± 8.89	39.57 ± 8.93	39.2 ± 9.58	38.1 ± 8.35	0.879
Post Bx-Pre Bx STAI-State changes ± SD	1.23 ± 8.36	4.23 ± 5.21	2.66 ± 11.18	−3.2 ± 5.52	**< 0.001**
Post-Bx Anxiety (VAS) ± SD	1.03 ± 1.34	1.17 ± 1.98	1.13 ± 0.73	0.8 ± 0.96	0.101
Post-Bx Pain(VAS) ± SD	2.58 ± 1.79	3.3 ± 2.1	3.1 ± 1.18	1.37 ± 1.3	**< 0.001**

M, mean; SD, standard deviation; bx, biopsy; STAI, State-Trait Anxiety Inventory; VAS, visual analog scale; bpm, beats per minute.

 Pairwise analysis showed that the conversation group differed significantly in post-biopsy VAS pain score, STAI-State score, and STAI score when compared to those of both the music and control groups. However, listening to music did not lead to a significant reduction compared to that in the control group (**Table 4**). 

**Table 4:** Pairwise comparisons of groups

**Post-Bx STAI-State**				
**Groups**	**Test Statistic**	**Std. Error**	**Std. Test Statistic**	**Sig.**
Conversation-Music	15.56	6.73	2.31	**0.02**
Conversation-Control	19.13	6.73	2.84	**0.004**
Music-Control	3.57	6.73	0.52	**0.596**
**Post-Bx pain**				
**Groups**	**Test Statistic**	**Std. Error**	**Std. Test Statistic**	**Sig.**
Conversation-Control	27.33	6.58	4.15	**< 0.001**
Conversation-Music	30.36	6.58	4.61	**< 0.001**
Control-Music	−3.03	6.58	−0.46	**0.645**
**Differences between pre and post-bx STAI-State**				
**Groups**	**Test Statistic**	**Std. Error**	**Std. Test Statistic**	**Sig.**
Conversation-Music	18.41	6.72	2.73	0.006
Conversation-Control	29.43	6.72	4.37	**< 0.001**
Music-Control	11.01	6.72	1.63	0.101

Bx, biopsy; STAI, State-Trait Anxiety Inventory.

 The patient opinion ("positive," "negative," or "abstained") on the repetition of the procedure was similar among the three groups. 

## DISCUSSION

 Previous studies evaluating different methods of reducing pain and anxiety during prostate biopsy reported varied outcomes. Some participants observed a significant reduction in anxiety scores, others in pain scores, whereas others in both scores. Conversely, certain methods have been shown to be ineffective in alleviating pain or anxiety during prostate biopsy. This study evaluated the effects of music, a method that has been explored in previous studies, and conversation, which has rarely been studied–on anxiety and pain in patients undergoing prostate biopsies. The findings revealed that listening to music did not reduce anxiety or pain; however, engaging the patient in conversations significantly reduced both. 

 Various techniques have been used to alleviate pain and anxiety during prostate biopsy. For instance, diaphragmatic breathing during TRUS-PBx significantly reduced anxiety, whereas hypnotherapy effectively reduced both pain and anxiety.^7,10^ The use of nitrous oxide (N_2_O) during TRUS-PBx had no impact on anxiety, but caused a slight reduction in pain levels.^27^ Similarly, patients who received video-based education before TRUS-PBx experienced lower anxiety levels than did those who only received verbal information. The use of augmented reality glasses during the procedure has also been shown to reduce pain.^8,9^ The results of this study revealed that conversion to TRUS-PBx significantly reduced both pain and anxiety, highlighting the potential of this simple, cost-effective, and accessible method for improving patient experience during prostate biopsy. 

 The effects of music have been investigated in several studies. A randomized controlled trial demonstrated that music had no significant difference in VAS and STAI scores compared to the control group.^18^ Two studies reported that music decreased the STAI-state state and VAS pain scores of patients undergoing TRUS-PBx.^17,19^ There are also studies in the literature showing that music only reduces STAI-State or VAS anxiety levels in patients undergoing TRUS-PBx and does not change pain level.^14-16^ The influence of listening to music during prostate biopsy is controversial; however, the findings of this study revealed that music has no effect on VAS pain, VAS anxiety, and STAI score. 

 The effect of music on hemodynamic parameters remains controversial. Two studies demonstrated no significant difference between the music and control groups.^18,19^ However, in other studies, significant differences were found in some hemodynamic parameters.^13-15,17^ The outcome of this study showed no significant difference in the hemodynamics of the patients. 

 The controversial findings in literature may depend on various factors such as the type of music chosen, use of headphones, volume level of music, and other variables. Reportedly, music can produce different clinical effects depending on its tempo.^28^

 In some studies, patient satisfaction and willingness to repeat the procedure were reported to be higher in the music group.^17,19^ However, in this study, there was no significant difference between the music and conversation groups. This may be attributed to the fact that this variable was recorded by using three categorical options instead of a continuous scale. 

 Packiam et al. indicated that crying or verbal distractions may be more effective than music. However, there is a lack of data on the effects of conversing with patients on pain and anxiety during prostate biopsy.^18^ Some studies have evaluated the impact of conversation and verbal distractions on pain and anxiety in other contexts. For example, pregnant women experience less pain during labor with distraction techniques such as conversation.^23^ The effect of conversation was compared with lidocaine/prilocaine cream in patients undergoing peripheral venous catheterization, and pain was found to be significantly lower in the conversation group.^22^ In another study, hand-holding combined with conversation was reported to be more effective than midazolam in reducing preoperative anxiety.^20^ Verbal distraction during burn dressing procedures was observed to alleviate anxiety but did not reduce pain.^29^ Similarly, a survey revealed that more patients preferred conversation as a distraction over receiving procedural information before venipuncture.^21^ Studies conducted with children have also shown that verbal distraction provided by parents significantly reduced pain during invasive procedures.^25^ This study is consistent with current data and demonstrated that engaging patients in conversation significantly reduced both pain and anxiety during TRUS-PBx. However, some studies have reported that conversation is ineffective in alleviating pain and anxiety. For example, conversation does not reduce pain in burn patients during rehabilitation or in patients presenting to the emergency department with acute pain.^26-30^

 The success of doctor-patient conversations depends on the selection of topics that the patient finds engaging and captivating. In this study, patients engaged in conversations about their interests and actively participated. During these conversations, the patients appeared to forget the procedure and distanced themselves from the pain and anxiety. 

 Although the study was planned as a prospective randomized controlled trial, it has limitations; due to the design, it was not a double-blind study. The pain level was assessed only before and after the procedure, whereas it should be assessed at each step of the process. The study was conducted at a single center with a relatively small sample size. In addition, the music was provided as ambient background music and not through headphones. 

## CONCLUSIONS

 This study demonstrated that listening to music has no significant effect on pain or anxiety in patients undergoing TRUS-PBx. Conversely, the findings showed that engaging patients in conversation significantly reduced pain and anxiety during the procedure. Distracting patients via conversation during the biopsy procedure increases patient comfort. However, further studies with larger sample sizes are necessary to validate and expand on these findings. 

## Data Availability

The data that support the findings of this study are available from the corresponding author, Volkan Selmi, upon reasonable request.
